# Supplementary material for: Causal interplay between lactose intolerance and gut microbiota: a combined bidirectional Mendelian randomization and in vivo validation study
Source: Front Nutr. 2026 Jun 1;13:1803337. doi: 10.3389/fnut.2026.1803337 (PMC13265576; doi:10.3389/fnut.2026.1803337)
Supplement: Supplementary file 1 [file Data_Sheet_1.zip › supplementary materials/Forward/leave one out cross validation/ebi-a-GCST90027786.finngen_R12_E4_LACTONAS.pdf]

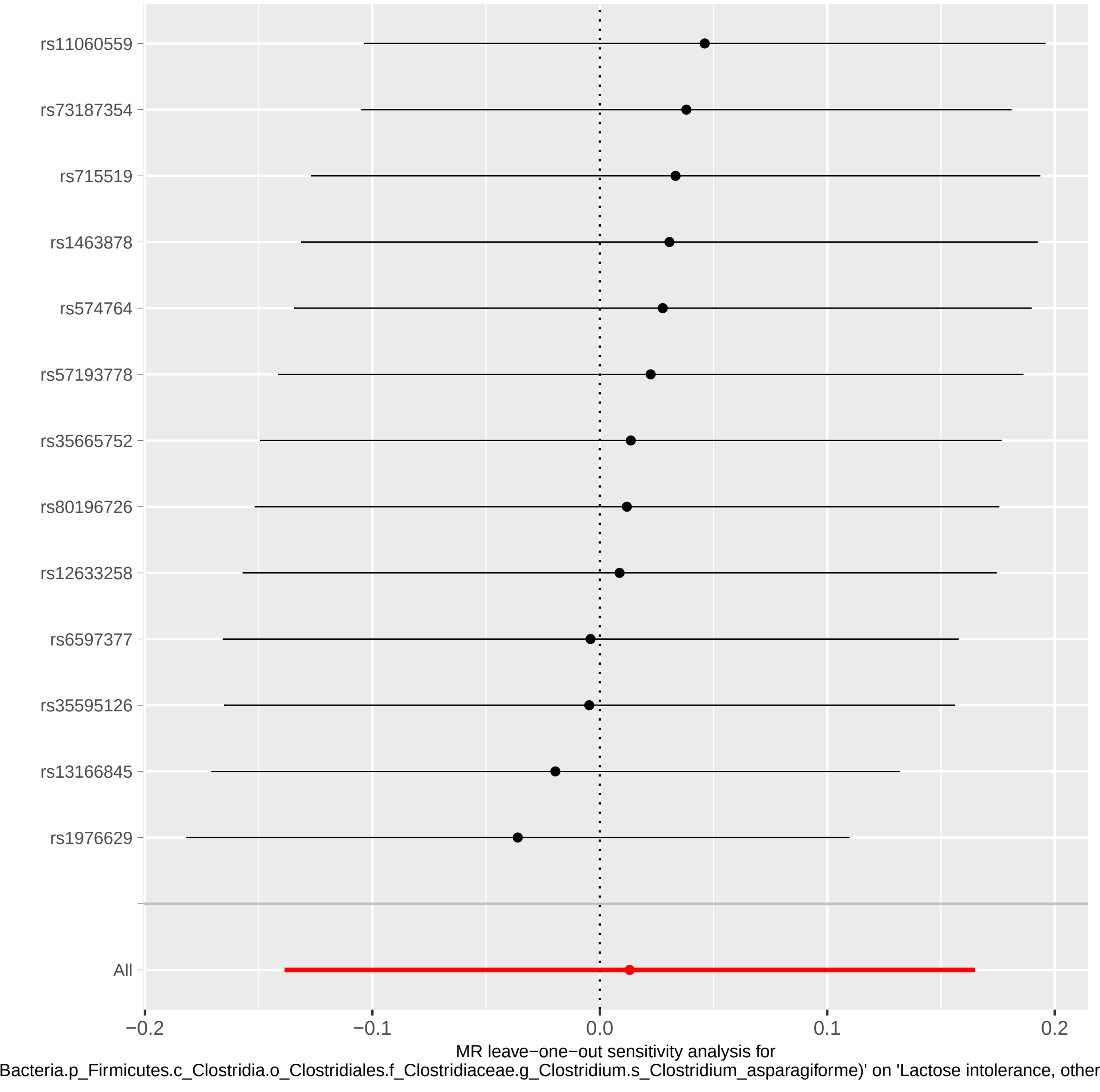
rs11060559
rs73187354
rs715519
rs1463878
rs574764
rs57193778
rs35665752
rs80196726
rs12633258
rs6597377
rs35595126
rs13166845
rs1976629
All
−0.2
−0.1
0.0
0.1
0.2
MR leave−one−out sensitivity analysis for
Bacteria.p_Firmicutes.c_Clostridia.o_Clostridiales.f_Clostridiaceae.g_Clostridium.s_Clostridium_asparagiforme)' on 'Lactose intolerance, other
